# Facilitating Development of Problem-Solving Skills in Veterinary Learners with Clinical Examples

**DOI:** 10.3390/vetsci9100510

**Published:** 2022-09-20

**Authors:** Amanda (Mandi) Nichole Carr, Roy Neville Kirkwood, Kiro Risto Petrovski

**Affiliations:** 1 Davies Livestock Research Centre, School of Animal and Veterinary Sciences, The University of Adelaide, Roseworthy, SA 5371, Australia; 2 School of Animal and Veterinary Sciences, The University of Adelaide, Roseworthy, SA 5371, Australia; 3 Australian Centre for Antimicrobial Resistance Ecology, School of Animal and Veterinary Sciences, The University of Adelaide, Roseworthy, SA 5371, Australia

**Keywords:** animal science, clinical activities, problem solving, teaching, veterinary learners

## Abstract

**Simple Summary:**

This review seeks to open discussion on the teaching of problem-solving skills in veterinary learners. Before a veterinary learn-er can solve a problem, they need to be able to recognize the problem. Then, information is gathered and economically viable solutions determined. To make problem-solving easier, we suggest a process with 5 elements: (1) define the problem list; (2) create an associated timeline; (3) describe the (anatomical) system involved or the pathophysiological principle applicable to the case; (4) propose management for the case; and (5) identify unique features of the case. In order to put the above into context, we end the review with an example case scenario showing the approach of teaching of problem-solving.

**Abstract:**

This paper seeks to open discussion on the teaching of problem-solving skills in veterinary learners. We start by defining the term problem before discussing what constitutes problem-solving. For veterinary medical learners, problem-solving techniques are similar to those of decision-making and are integral to clinical reasoning. Problem-solving requires the veterinary learner to organize information logically to allow application of prior or new knowledge in arriving at a solution. The decision-making must encompass choices that provide the most beneficial and economical approach. In a modification of an existing protocol, we suggest the inclusion of the 5 elements: (1) define the problem list; (2) create an associated timeline; (3) describe the (anatomical) system involved or the pathophysiological principle applicable to the case; (4) propose management for the case; and (5) identify unique features of the case. During problem-solving activities, the instructor should take the role of facilitator rather than teacher. Skills utilized in the facilitation of problem-solving by learners include coaching, differential reinforcement, effective feedback, modelling and ‘think out loud’. Effective feedback must inform learners of their progress and performance, as this is fundamental to continued learning and motivation to succeed. In order to put the above into context, we end with an example case scenario showing how we would approach the teaching of problem-solving to veterinary learners.

## 1. Introduction

One of the relatively common deficiencies in veterinary medical education is the lack of assisting of veterinary learners in the development of their clinical reasoning skills. However, if successful in the development of these skills it will make them more competitive in the employment market, an important outcome for modern education [1,2]. Indeed, developing clinical reasoning skills in learners is a requisite of any accreditation body for schools that are required to undergo accreditation [3]. For example, the Royal College of Veterinary Surgeons accreditation is based on Day One Competencies, and clinical reasoning and problem-solving competencies are clearly stated in the document (Competencies 22–25) [3]. From an educational perspective, clinical reasoning may be seen as skills in both complex problem resolution and a critical thinking. Utilizing case-based scenarios will provide suitable opportunities to assist veterinary learners in the development and implementation of their clinical reasoning. Solving of case-based scenarios should assist them in developing new skills of which, at a minimum, should stimulate self-directed (‘deep’) learning [4,5]. The case-based scenarios should be carefully chosen with the aim to encourage learners to incorporate knowledge from a variety of backgrounds, including basic, preclinical and clinical sciences [4].

Clinical reasoning during a clinical encounter consists of a few general steps: (1) collecting information; (2) analysis of the information; (3) management of the case; and (4) opportunistic promotion of animal health and welfare [6,7,8,9,10,11]. During each of these steps, the collected information may present elements that need to be solved (problem). For a positive outcome for the case, clinical reasoning should satisfactorily address each problem encountered.

While the human medical field provides pertinent literature concerning the development of problem-solving skills in medical learners [4,12,13,14], there is a dearth of such literature concerning veterinary learners with exceptions in some areas where emerging literature is available such in communication that includes some basic problem-solving skills. The present paper seeks to start to address this lack of literature related to teaching problem-solving skills related specifically to the veterinary medical field. In our current OneHealth world encompassing environmental, plant, animal and human health, and believing that teaching approaches are a transferrable skill, we will re-examine some human literature through a veterinary lens, and reinforce it with a veterinary clinical example. However, before addressing this issue, we must first define the meaning of the term problem and, more importantly, the nature of problem-solving.

## 2. Problem and Problem-Solving

### 2.1. What Is a Problem?

The term problem is difficult to clearly define and a plethora of explanations of the meaning of this term exist. Most common dictionary-based explanations related to problems in veterinary medicine would include something along the lines of a matter or situation regarded as unwelcome or harmful and needing to be dealt with and overcome. Problems may be ill-defined or well-defined. Problems with discrete presentation and clearly identifiable solutions are referred to as well-defined while problems with unclear presentation and/or many potential solutions are referred to as ill-defined [13].

### 2.2. What Is Problem-Solving

**Figure 1:**
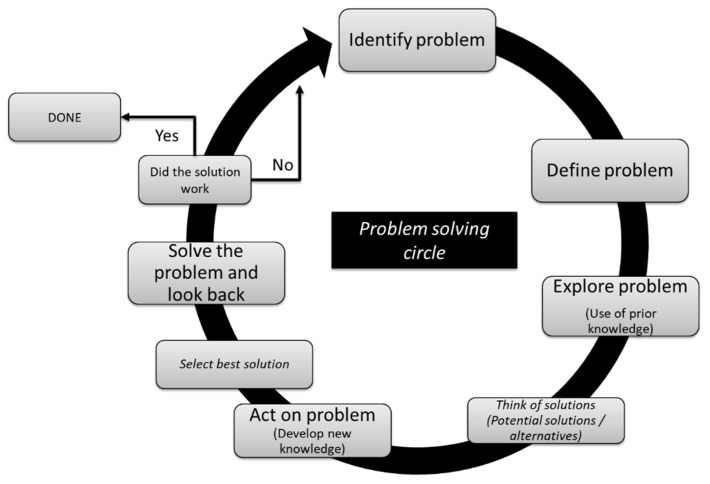
Problem-solving circle related to a veterinary medical problem.

Problem solving should encourage the use of higher-order thinking skills to effectively manage and critically determine realistic solutions to problems. It is a basic skill or tool that is needed by employees for addressing workplace demands [1,15]. Problem-solving requires a complex set of attitudinal, behavioral and cognitive components, often used in a multiple-step process to arrive to the solution (i.e., collection, interpretation and integration of information) [14,16]. During the process of problem solving, a learner needs to combine old rules (cognition) and form new rules (cognition and metacognition; Figure 1). Each step in problem-solving can be seen as a decision tree [17] and may be approached using alternate pathways [18]. The pathway taken by the learner is usually one they have learned previously or a slight modification of it [18], and their personal preferences acquired during their studies and life experiences. Considering that problem-solving is only partly dependent on the context, problem solving skills can be utilized in future different and similar encounters [18]. For veterinary medical learners, problem-solving techniques are, in essence, similar to those of decision-making and are a key competence of learners/practitioners that is essential in clinical reasoning.

Problem-solving should encourage learners to organize information in a logical manner [4] to allow them to apply a variety of prior learning (cognition) and new knowledge (metacognition) in the process of reaching a solution [4,13,15]. This should be accompanied by self-directed learning, including active listening, critical thinking and reflective practice [4,13].

### 2.3. What Is Decision-Making?

**Figure 2:**
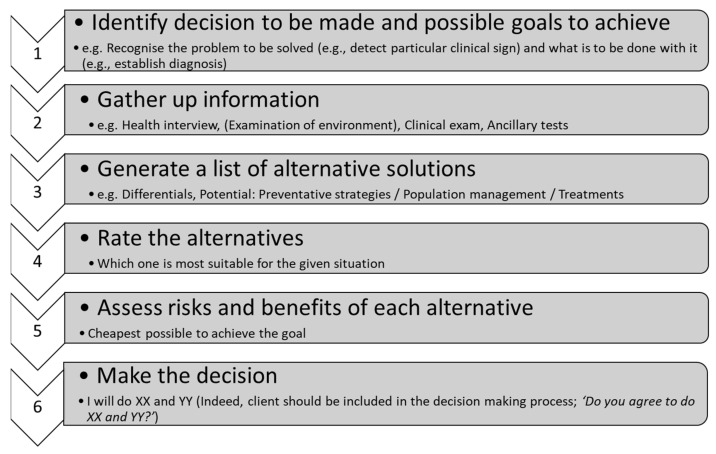
Decision-making process applicable to veterinary medical problem.

Decision-making requires a veterinary learner/practitioner to decide which course of action/issue/solution to pursue (extrapolated from [6,19]). The number of choices may be small or large. The decision-making process should select (compare and contrast) which choice would be most beneficial, economical and satisfactory for that particular situation for all involved parties, the client, the patient and the veterinary clinician (Figure 2). 

## 3. Teaching Problem-Solving

### 3.1. Problem-Solving Techniques

A plethora of problem-solving techniques have been described in a variety of scientific areas [4,16,20], including mathematics [21,22] and medical education [23]. In short, any teaching of problem solving is better than doing nothing, with the expectation that the learner will appreciate an ongoing need for further training [16,24,25]. This may be delivered as a specifically designed professional course or be adopted as a teaching principle within many disciplines. Problem-solving is discipline specific [20,22]. For medical education, it seems that a partially heuristic approach to problem-solving is acceptable [26]. Exposure to explicit instructions in problem solving has been reported to improve these skills in learners [4,15,16,18,23,27]. Explicit instruction should provide the learner with a ‘protocol’ (usually a decision tree) that can be applied to the problem solving [4,15,17]. Two frequently cited frameworks for teaching clinical reasoning are Clinical Reasoning Cycle [27,28] and Clinical Judgment Model [27,29]. However, for the problem-solving exercise, simplified problem-solving protocols may be more applicable.

Each step in the explicit instructions for problem-solving should lead to a maximum of 3–6 branches of a particular decision tree. If there is a likelihood that a particular step would result in more than 6 branches, it is advised that the particular step is further branched into smaller branches (steps) of the explicit instructions [17]. Then, this protocol can be applied to other scenarios [25]. The ‘protocol’ is often broadly discipline-dependent (e.g., computer software programming versus medical education) [19]. For the successful solving of the problem, the learner must approach the solving relying on some prior knowledge [18,21]. This is very important to note for learners in any of the medical education areas, including veterinary medicine since, without prior knowledge, problem-solving will be difficult or even impossible [30]. Hence, problem-solving activities must be tailored to the level of development of the learner (e.g., when learners are classified using the (O)RIME framework, different cases/clinical encounters are required for learners in R(eporter) compared to M(anager) levels) [31].

Meyer et al. [3] have proposed a problem-solving protocol applicable to case-based scenarios for medical disciplines that is composed of 4 elements: (1) define unfamiliar terms; (2) create a timeline associated with the problem; (3) describe the (anatomical) system involved; and (4) identify any unique features associated with the case. This protocol may be suitable for stimulation of deep learning in various disciplines and may be applicable to problem-solving in case-based scenarios related to clinical encounters. However, for applicability to clinical encounters, we propose changing the first element to ‘define the problem list’. As not all case-based scenarios can be solved by anatomical location (e.g., a problem related to a particular pathophysiologic principle or sign or syndrome, such as cough, diarrhea or mastitis), we propose the third element can be either what Meyer et al. [3] proposed, or be extended to ‘describe the (anatomical) system involved or the pathophysiological principle applicable to the case’. Finally, for applicability to case-based scenarios related to clinical encounters, the protocol needs a fifth element, which we propose to be ‘management for the case’. This element may address further data gathering and/or treatment and/or prevention of future cases of similar character.

Hence, our modified protocol applicable to clinical encounter-based case scenarios would include the following 5 elements: (1) define the problem list; (2) create a timeline associated with the problem; (3) describe the (anatomical) system involved or the pathophysiological principle applicable to the case; (4) propose management for the case; and (5) identify any unique features associated with the case. 

### 3.2. Specific and Non-Specific Problems

Some problems detected during a clinical encounter are specific (distinguishing), others are not [32,33]. Specific problems, such as loss of menace response, mucus in feces or a swollen joint, assist the learner in making the decision (e.g., diagnosis). Non-specific problems such as pyrexia or loss of appetite, are less informative, or even non-informative, in the decision-making process. Making learners aware of the difference between specific and non-specific problems is very important [33] as this will allow them to minimize the amount of information handled at any given time.

### 3.3. Role of the Instructor in Teaching Problem-Solving

A problem in veterinary medical education is the frequent lack of educational training of the veterinary instructors [2,34,35,36]. Indeed, one of the medical areas mentioned as hugely deficient in 1970s was the instructor’s lack of educational training in problem-solving [37], and it remains true today. 

The instructor should take more of the role of facilitator rather than teacher. Skills than can be utilized in the facilitation of problem-solving by learners include, but not limited to, coaching, differential reinforcement, effective feedback, modeling and ‘think out loud’ [17,26]. Effective feedback is essential to deep learning in medical, including veterinary medical, education [23,26,38]. Learners must be informed of their progress and performance as this is fundamental to continued learning and the motivation to succeed [38]. Learners should be stimulated to self-assess (i.e., reflect) on problem-solving activities as that is also essential to deep-learning [27].

### 3.4. Learner Benefits from Learning Problem-Solving Skills

The benefit of learning problem-solving skills is that they can then by applied to other similar or dissimilar problems encountered in clinical practice and, potentially, also in everyday life [2,4,7]. Once learned and repeatedly applied, problem-solving should become habitual and easily expanded [17]. The decrease in clinical errors after graduation is one of the outcomes that has been mentioned as an important benefit for learners [35]. The knowledge of the risk of introduction of false negative/positive test results should also benefit learners as it should decrease their reliance on laboratory data in the decision-making process [39]. 

## 4. Addressing Various Elements of Inadequate Problem-Solving

A learner may lack elements necessary for effective problem-solving during a clinical encounter (Table 1; [11,12,26,27,33,35,40,41,42,43]). Similarly, this would also apply to case-related discussions, particularly when working in smaller groups. We do note that this table is not holistic and some areas are not addressed (e.g., a learner with inappropriate behavior and/or family/personal problems and/or lack of fitness to practice and/or lack of recognition of a learning opportunity and/or lack of self-esteem). We acknowledge the importance of these areas, however, they are beyond the scope of this writing. Although not a focus of the present paper, another important aspect that should be mentioned is the relative lack of general training of ‘soft skills’ in veterinary programs, including but not limited to communication, emotional intelligence, inter-personnel skills and professional behaviors. This is to the detriment of the teaching process when clinical disciplines are introduced. Therefore, learners do not know how to handle their emotions, stress, time pressure, being assessed and then even the best prepared didactic methodologies, as those presented in this paper fail to work. This writing concentrates on learners having problems with application of knowledge. For learners with problem-solving difficulties, they may show insufficient progress or may have difficulties with a particular problem-solving skill [43].

We recognize that addressing problem-solving difficulties also requires some practice and experience by the instructor, inclusive of specific training. The starting point in addressing the difficulties in problem-solving, from an instructor’s view, is the recognition of the different types of difficulty in a learner. Based on this, appropriate intervention strategies can be implemented. Effective feedback should be used to assist the learner in developing better problem-solving clinical skills as part of developing appropriate clinical reasoning. We will use an example of guiding the clinical teaching of learners using the five microskills model [35,44,45]. Most of the elements lacking during the problem-solving can be addressed during the ‘Correct mistakes’ microskill. However, some elements may be addressed earlier, in the ‘Probe for supportive evidence’ and, less likely, the ‘Get a commitment microskill (provided instructors observe the learner during data collection).

### Poor Problem Detection during Clinical Problem-Solving

A cornerstone of problem-solving is problem identification/recognition [24]. However, in veterinary medical education, anecdotal evidence exist that some learners do not recognize some problems (as observed by the authors). Indeed, the lack of problem detection is also reported in other disciplines [6,24]. Based on literature evidence [6,14,26,42,46,47,48], a lack of recognition of problems may partly be due to:

- Individual capacity and differences in mental problem-solving skills between people
- Differences in age and gender (variable reports; detected during exams but also in clinical practice) with females and more mature people showing usually better problem-solving skills
- Flows in data gathering, with people having problems in data gathering having poorer problem-solving skills
- Following strict guidelines with little to no adjustments to the particular circumstances/presentations, seeking ‘textbook’ answers and so failing to realize that complaints often do not express themselves clearly such as with poorly expressed signs and syndromes
- Lack of connection of involvement of multiple organ systems/pathophysiology
- Lack of capacity to deal with multiple activities and hypotheses at once (multitasking)
- Lack of lifelong learning/continuing education of veterinary instructors adversely impacting availability of current information and so effective teaching
- Learner inexperience in problem-solving in veterinary medical fields, but may be also generalized poor problem-solving

Therefore, prior to seeking solutions, problem-solving should start with helping the learner to identify/recognize the problem. One of the possible approaches to the lack of problem recognition is so-called ‘component opportunity’ [24] where the ‘bigger problem’ is split into smaller components, each with at least one ‘problem’ that needs solving. Due to the differences between people in the mental skills required to solve the ‘bigger problem’, splitting it into components is often more easily said than done as the instructor would need to be able to facilitate the learner to do this on their own. 

## 5. Example Clinical Encounter

The following information is provided to the learners

- Case encounter occurs early morning
- Holstein-Friesian calf from an embryo transfer recipient cow
- Delivered 10 days ago without assistance
- Had high vigor until last night
- Sudden onset of diarrhea about 6- 8 h before being seen
- Now in sternal recumbency, semi-obtunded
- Examination findings
  ○ Cardinal signs all at the lower end of ‘normal’
  ○ Mucous membranes tacky but relatively moist
  ○ Skin tent 8 s
  ○ Eyes sunken about 0.5 cm from the orbit
  ○ Lack of suck reflex
  ○ No alimentary sounds at the abdomen

### 5.1. What Should Problem-Solving Address?

Using our protocol, modified from Meyer et al. [3], the problem-solving should address the following

(1) **Construct the problem list**
  - What is missing on the learner’s problem list
(2) **Create a timeline associated with the problem**
  - 10 days old
    - Can exclude relevant congenital problems
  - Was OK up to last night
  - Diarrhea detected some 6–8 h before the encounter
    - Peracute to acute case
    - Laboratory findings may be unreliable
(3) **Describe the (anatomical) system involved or the pathophysiological principle applicable to the case**
  - Alimentary
    - Loss of absorptive capacity
    - Mechanical damage to the intestinal wall
    - Overgrowth of unwanted bacteria and production of D-Lactate
  - Cardiovascular
    - Dehydration
    - Hypokalemia
    - Metabolic acidosis
      1. Neonatal calf diarrhea usually results in a metabolic acidosis, not a metabolic alkalosis
    - Toxemia
      1. May not be high on the list due to the lack of the toxic line on the gums
  - Hemolymphatic
    - Dehydration and hypovolemic shock
    - Sepsis absent or compensated, plus toxemia
    - Toxemia likely absent
  - Nervous
    - Dehydration
    - Excess D-Lactate
    - Hypoglycemia
    - Toxemia
  - Respiratory
    - Metabolic acidosis
  - Urinary
    - Lack of perfusion
(4) **Propose management for the case**
  - Immediate
    - Treatment should address important derangements
      1. Dehydration
      2. Hypoglycemia
      3. Hypokalemia
      4. Metabolic acidosis
      5. ±Toxemia
    - Route of administration of the fluid therapy should be considered
      1. Initially, intravenous due to lack of suck reflex and inability to absorb oral fluids
      2. Continue with oral
    - Fluid type
    - Treatment plan
      1. Bolus in 1st hour
      2. On-going
  - On-going care
    - On-going fluid therapy
    - Re-introduction of milk
  - Prevention of further case
    - Caused by infectious or non-infectious cause
      1. All undifferentiated calf diarrhea should be considered infectious until proven otherwise
      2. If infectious
        - Contagious or not
        - Zoonotic or not
    - Colostrum management
    - Housing management
  - Zoonotic risk
    - Consider immune-compromised people
(5) **Identify any unique features associated with the case**
  - Some of the acid-base (e.g., the excess of D-Lactate) and electrolyte imbalances (e.g., apparent hyperkalemia when the body suffers from absolute hypokalemia) may not be detected by laboratory tests
  - Due to the very rapid development, some of the clinical findings may be unreliable (e.g., the real degree of dehydration may not be properly detected with estimation of enophthalmia and/or skin tent)

### 5.2. Assisting the Learner to Recognise Problems with Example Discussions

We provide a variety of examples of potential presentations of the learner’s problem-solving skills and, for each of the presentation, the potential advisory discussion to assist the learner in addressing the lack of problem-solving skills (Table 2).

## 6. Conclusions

The teaching of effective problem solving is essential to ensure the clinical competence of veterinary learners. From the literature we have reviewed, we conclude that learners should be guided through 5 elements, namely define the problem list, create a time line for the problem, describe involved anatomical system/pathophysiology, propose case management, and identify unique case features. In common with all clinical teaching, while guiding learners through these elements the provision of appropriate feedback is required. If all this is done effectively, the developed problem-solving skills will be transferrable to other similar or dissimilar cases and even into everyday life.

## 7. Glossary

**Case-based discussion**—clinical teaching tool that consists of a structured approach to learners’ clinical reasoning around written case records and/or structured interview with a simulated client and patient.

**Clinical encounter**—any physical or virtual contact with a veterinary patient and client (e.g., owner, employee of an enterprise) with a primary responsibility to carry out clinical assessment or activity.

**Clinical instructor**—in addition to the regular veterinary practitioner’s duties, a clinical instructor should fulfil roles of assessor, facilitator, mentor, preceptor, role-model, supervisor, and teacher of veterinary learners in a clinical teaching environment. Apprentice/intern in the upper years, Resident, Veterinary educator/teacher, Veterinary practitioner.

**Clinical reasoning**—process during which a learner collects information, process it, comes to an understanding of the problem presented during a clinical encounter, and prepares a management plan, followed by evaluation of the outcome and self-reflection. Common synonyms include clinical acumen, clinical critical thinking, clinical decision-making, clinical judgment, clinical problem-solving, and clinical rationale.

**Critical thinking**—self-directed, self-disciplined, self-monitored, and self-corrective objective, unbiased analysis and evaluation of information form a variety of sources in order to form a judgement.

**Deep learning**—aiming for mastery of essential academic content; thinking critically and solving complex problems; working collaboratively and communicating effectively; having an academic mindset; and being empowered through self-directed learning.

**Differential reinforcement**—process in which a specific response is reinforced or not (i.e., positive or negative reinforcement) in a particular context with aim to address challenging of undesirable behavior.

**Five microskills model**—Instructor-centered model of clinical teaching: (1) Get a commitment; (2) Probe for supporting evidence; (3) Teach general rules; (4) Reinforce what was done well; and (5) Correct mistakes.

**Heuristic**—general methods used in problem-solving, applicable to problem in any content domain.

**Higher order thinking skills**—A series of competencies that can be utilized in analysis and evaluation of complex information, including categorization, manipulation, and connection of facts, conceptualization, troubleshooting for solutions and problem-solving. It is on a level that is higher than memorizing facts or recollection only.

**Metacognition**—This is a critical awareness of one’s thought processes and learning, and an understanding of the patterns of thinking and learning.

**Self-directed learning**—learners take charge of their own learning process by identifying learning needs, goals, and strategies and evaluating learning performances and outcomes. Learner-centered approach to learning.

## Figures and Tables

**Table 1:** Addressing common elements of inadequate problem-solving by veterinary learners.

Element Lacking/Parameter	Detection	Strategy to Assist the Learner	Example of Facilitating Discussion
Complex approach to a problem	Reflection ‘Think aloud’	Purposeful questioning Team work	“Could we consider different ways to solve this problem?”
Inefficient data collection	Omission errors (e.g., Procedure/Process incomplete; Sticks to standardized protocol, disregarding clinical presentation)	Modelling of data collection followed by solicitation of new data presentation by the learner Providing more opportunities to practice (e.g., use of simulator/s) Purposeful observation of learners who are skilled in the particular omission	“Can we again go through the process of data collection in this case? I would like you to observe me doing the health interview and clinical exam. I would like you to think of what additional information or clarification would be helpful in this particular case that you may have omitted in your data collection.”
	Presentation incomplete (e.g., Disregarding data judged as not important, Lack of identification of what is important)	Modelling of data collection followed by solicitation of new data presentation by the learner Providing more opportunities to practice	“Can we again go through the process of data collection in this case? I would like you to observe me doing the health interview and clinical exam. You should pay attention to the items used in obtaining information. Then we can re-discuss your findings.”
Inefficient data reporting	Inability to create appropriate/prioritized list of differential diagnoses	Increased clinical exposure and requirement to present cases at rounds Modelling of data analysis and creating differential diagnosis list Purposeful questioning Providing more opportunities to practice Providing regular feedback ‘Think aloud’	“Can you summarize what the main findings are in one to two sentences?” or “Can you write down the list of problems for this case?” **Learner does**. “Now, it will be good to hear a prioritized list of differentials considering rule-ins and rule-outs applicable to this case.” or “Please prioritize the list of problems detected in this case to assist us in creating an appropriate list of differentials.”
	Reporting/Summarizing Not open to new problem relationships Only loosely connected to the encounter		“Let’s discuss further the data gathered. I think you summarized the case you presented as a calf with a fever, swelling of multiple joints, which are tender and, from the health interview you know that this calf had an episode of diarrhea some two weeks ago. In fact, that would be a very good summary. However, you also added a few elements which are very loosely connected to this case. For example, yearlings having diarrhea that seems to be related to parasitic burden. This led you to a conclusion that this calf may be suffering from parasitic gastroenteritis. Parasitic gastroenteritis may be the problem of yearlings which are on pasture. However, this 18-day old calf has been hand-reared. I would like you to think of two completely separate problems. Can we re-discuss your differential again?” **or** “Please watch me discussing this with the client, particularly how I would put things together.”
	Seek answer in ‘textbook’ presentations only		“Let’s discuss further the data you gathered. Now, it will be good to think of which diagnoses may fit some or a majority of these problems, although they may not be an exact match.”
Poor problem detection	Difficulty seeing the scope of a problem Lack of recognition of a particular clinical problem Lack of recognition of the effect of the problem on the client	Employ ‘Component opportunity’ Purposeful questioning	“Let’s discuss further the data gathered. You mentioned that in this cow you detected a fever, cough, purulent nasal discharge, abnormal lung sounds on the right cranio-ventral side and that she has calved some 7 days ago. You also mentioned that the client has given her oral calcium about 2 days after calving for suspected milk fever. You connected all detected clinical signs but skipped through the health interview information in your interpretation. I think, it is important to consider the health interview in your problem presentation. Can you think of the main pathophysiological mechanism of hypocalcemia and how that may be related to the current case?”
			“Do you notice anything else that should be on the list of problems for this particular case?”
			“Can we discuss the parameters you detected and list them separately into ‘normal’ and abnormal? We are all aware that these should be considered together with other findings but for the purpose of this exercise let’s consider them independently. Do you agree?”
Poor implementation of solutions	Lack of tailoring the solution to the particular circumstances/presentation	Purposeful questioning Modelling of Communication skills Selection and implementation of solution/s ‘Think aloud’	“Let’s discuss further the management you propose. You mentioned that this dairy enterprise has been under new management for a few months and there has been a rise in cases of left displaced abomasum. Can you think of management solutions for this enterprise that are a priority to prevent future cases from occurring rather than continue with surgical interventions ?”
	Lack of suitable solutions (e.g., learner lacks understanding of what is the goal of the intervention)		“Can we go through the solution you are proposing? This farm has an individual cow with respiratory disease and you have recommended treatment with ceftiofur, a third-generation cephalosporin antimicrobial. I think it is important to consider responsible antimicrobial use in your treatment recommendation. Can you think of another antimicrobial that may be more suitable for an individual case and how that may be related to good antimicrobial stewardship?”
	Proposed solution offensive		“I think we should discuss how you delivered your recommendations to the client? You mentioned that his management was poor and all his problems were due to this. It is important to consider how that made the client feel. We are aware that there are some management areas that need to be improved. However, the delivery of the message must be carefully crafted. It is important to praise the client for what they are currently doing well but also make suggestions on how to improve the current practices. Can you think of a way of doing this?”
	Seeks ‘textbook’ solutions only		“Let’s discuss further the solutions you propose. It is good to think of tailoring solutions for this particular enterprise, even if they are not an exact match to each individual problem occurring on the farm.”
Poor evaluation of the outcome	Lack of holistic approach to the expected outcome		

**Table 2:** Using an example of undifferentiated calf diarrhea in assisting veterinary learners with problem recognition and the specific roles of each problem in the entire problem presentation during a case-based discussion or a clinical encounter.

Item	Potential Causes/Importance	Example Assisting Discussion
***Clinical problems that should be detected***		
Cardinal signs ‘normal’	No sepsis, but may have toxemia	
Enophthalmos 0.5 cm	Used to estimate dehydration	“It would be useful to think of pathophysiologic mechanisms that may lead to enophthalmos.”
Lack of alimentary motility	Used to estimate D-lactate acidemia and hypokalemia	“Can you think of few factors that may result in decreased motility of the alimentary system (ileus) in this calf?”
Lack of suck reflex	Used to estimate D-lactate acidemia and hypokalemia	“Can you think of few factors that may result in loss of the suck reflex in this calf?”
Semi-obtunded	Acidemia Excess D-lactate Dehydration Hypoglycemia Hypokalemia Toxemia	“If we know that lab results for some metabolites and electrolytes may not be reliable, we have to think about what may be the effects of some of these metabolites or electrolytes on the calf’s demeanor and on the parasympathetic system.” “Can you also think of what may be happening with the gut microbiota and what may be the effect of the changes if they occur?”
Severe peracute diarrhea	Rapidly developing	*After discussion on enophthalmos and skin tent: *“We have discussed the significance of the enophthalmos and the prolonged skin tent. Think of the speed of development of these signs and how they may be affected by the peracute development of diarrhea in this calf?”
Skin tent 8 sec	Used to estimate dehydration	“It would be good to think of the pathophysiologic mechanisms that may lead to prolonged skin tent.”
Sternal recumbency	Acidemia Dehydration Hypoglycemia Toxemia	“Can you think of a few factors that may result in sternal recumbency in this calf?”
***Laboratory findings that need to be considered***		
Hypokalemia		
Metabolic acidosis	Altered demeanor Altered respiratory rate	“We know that typically, diarrhea leads to alkalemia due to loss of electrolytes in feces. Indeed, this is true for mature cattle as well. However, this may not be the situation in neonates. Can you think of what electrolytes may be lost with the diarrhea in this calf? Then, please think of the outcome on the acid-base status and the expected electrolyte balance?”
Unreliable laboratory findings	Laboratory tests should be interpreted cautiously D-lactate not routinely measured Potassium shifts extracellular (apparent hyperkalemia in real hypokalemia)	“It is important to realize that due to a few pathophysiological derangements, blood electrolytes and acid-base balance testing may not be reliable in neonatal calves. Can you think what electrolytes may not be represented correctly in the biochemistry panel in this calf and why?” “Can you think of what may be happening with the gut microbiota and what may be the effect of the changes, if they occur?” *Learner should answer that there is overgrowth of gram-negative bacteria and production of D-Lactate.* “Now, can you think of what form of lactate is normally produced in the body of mammals that is a subject of the laboratory analysis?” *Learner should answer L-Lactate.* “Now, after we established these differences, could you think of reasons why D-Lactate concentrations not been reported with the biochemical tests?” “Some electrolytes in diarrhea shift from intracellular to extracellular. Can you give an example?” *Learner should answer potassium,*“Can you now think what would be the result of this shift of potassium on the serum biochemical test results?”
***Additional considerations in the management of this case***		
10-day old calf	Age-related etiology	“Can you think of the common causative agents associated with calf diarrhea?” *Learner lists a few. *“Now, can you think of the age at which these causative agents usually cause diarrhea in calves?”
Dairy	Not on dam	“Let’s think of the usual management of neonatal dairy calves. Can you think on the feature that differs from beef calves?”
Embryo-transfer product	Expensive	“Let’s think about the cost-benefit of treatment of this calf. Can you think what parameters should be accounted for before making the final decision?”
Prevention of further cases	Animal welfare Client frustration Economics Loss of genetics	“Can you tell me what you think should be done in future to prevent recurrence of this problem? What factors should be considered when making decisions on the prevention strategies? Can you explain why prevention is important for the client?”
Role of management	Colostrum management Environment Hygiene Stress	“Could you explain what management factors may contribute to the occurrence of diarrhea in this calf on this enterprise?”
Zoonotic potential	Public health	“We already mentioned the common pathogens associated with calf diarrhea. Can you think of what risks there are for people involved in management of this and other calves on the enterprise and discuss what specific factors need to be considered?”

## Data Availability

Not applicable.
